# Aphasic Syndromes in Cerebral Venous and Dural Sinuses Thrombosis—A Review of the Literature

**DOI:** 10.3390/life12111684

**Published:** 2022-10-24

**Authors:** Georgiana Munteanu, Andrei Gheorghe Marius Motoc, Traian Flavius Dan, Anca Elena Gogu, Dragos Catalin Jianu

**Affiliations:** 1 First Division of Neurology, Department of Neurosciences, “Victor Babeș” University of Medicine and Pharmacy, 300041 Timișoara, Romania; 2 Centre for Cognitive Research in Neuropsychiatric Pathology (NeuroPsy-Cog), “Victor Babeș” University of Medicine and Pharmacy, 300736 Timișoara, Romania; 3 First Department of Neurology, “Pius Brînzeu” Emergency County Hospital, 300736 Timișoara, Romania; 4 Department of Anatomy and Embryology, “Victor Babeș” University of Medicine and Pharmacy, 300041 Timișoara, Romania

**Keywords:** aphasia, cerebral venous thrombosis, intracranial dural sinus thrombosis, CVT

## Abstract

Aphasia is an acquired central disorder of language that affects a person’s ability to understand and/or produce spoken and written language, caused by lesions situated usually in the dominant (left) cerebral hemisphere. On one hand aphasia has a prevalence of 25–30% in acute ischemic stroke, especially in arterial infarcts. On the other hand, cerebral venous and dural sinuses thrombosis (CVT) remains a less common and underdiagnosed cause of ischemic stroke (0.5–1% of all strokes). Aphasia has been observed in almost 20% of patients who suffered CVT. The presence of aphasia is considered a negative predictive factor in patients with stroke, severe language disorders corresponding to arduous recovery. Taking into consideration data from the literature, aphasia is also considered a predictive factor for patients with CVT; its absences, together with the absence of worsening after admission, are determinants of complete recovery after CVT. This review has as the principal role of gathering current information from the literature (PubMed database 2012–2022) regarding the clinical features of aphasic syndromes and its incidence in patients with CVT. The main conclusion of this review was that aphasic syndromes are not usually the consequence of isolated thrombosis of dural sinuses or cerebral veins thrombosis. The most frequent form of CVT that determines aphasia is represented by the left transverse sinus thrombosis associated with a posterior left temporal lesion (due to left temporal cortical veins thrombosis), followed by the superior sagittal sinus thrombosis associated with a left frontal lesion (due to left frontal cortical veins thrombosis). Only a few cases are presenting isolated cortical veins thrombosis and left thalamus lesions due to deep cerebral vein thrombosis. We also concluded that the most important demographic factor was the gender of the patients, women being more affected than men, due to their postpartum condition.

## 1. Introduction

On one hand, aphasia, which is an acquired central disorder of language, is one of the most common focal neurologic deficits observed in CVT, being noticed in 19–24% of cases [1,2]. On the other hand—CVT represents a less common and underdiagnosed cause of stroke (0.5–1% of all strokes) [3]. Unlike older studies that showed an incidence of 0.2–0.5/100,000 person/year [4], recent studies show a higher incidence (1.32–1.57/100,000 person/year) [5]. This might be the consequence of greater accessibility to imaging technologies as well as an increased interest and awareness in diagnosing CVT. Aphasia is usually the consequence of left lateral sinus (LS) and tributaries cortical veins thrombosis from the dominant hemisphere. In 2009, Damak et al. conducted a study with 195 patients with CVT. Among those patients, 157 (80.51%) suffered from lateral sinus thrombosis. The researchers evaluated the percentage of patients with lateral sinus thrombosis who had suffered aphasia and concluded that ~13% were presenting language disturbances, being the most common focal sign [6]. The presence of aphasia in stroke is well known as being a negative predictive factor in the evolution of patients: the more severe the language disorder, the more difficult the recovery of these patients. Furthermore, Ferro et al. concluded in one of theirs studies in 2002 that the determinants of complete recovery after CVT were: the absence of aphasia and the absence of worsening after admission [7].

CVT is a special category of cerebrovascular disease, encountered especially in children and younger adults rather than in arterial ischemic strokes (frequently in those with pro-thrombotic conditions, such as inherited thrombophilia), mostly women (oral contraceptives, pregnancy, postpartum or post-abortion). Early acquaintance of CVT’s clinical features (including language disturbances) may improve the prognosis of this potentially fatal pathology.

### 1.1. Definition of Aphasia

The term of aphasia includes the inability of a person to understand and/or produce spoken language, sometimes also accompanied by the incapacity of reading (alexia) and writing (agraphia). It is always the consequence of an acquired central disorder, meaning that it occurs after the language has already been developed. Aphasia should be differentiated from a peripheral disorder of language (such as weakness or discoordination of phonatory musculature) that may easily simulate aphasia [8].

### 1.2. Language Localization

The organization of language networks has not been yet fully elucidated, despite the technological advances made in past years. Using complex techniques, especially functional neuroimaging, scientists have proven that language production is the result of neural activation from a vast network scattered in different structures of the brain: the cortex, the basal ganglia, the cerebellum and even the brainstem. This is the reason a disruption of this network determines spectrum of clinical signs. Functional neuroimaging studies suggested that the core of language operates in the left perisylvian regions in most individuals (95% right-handed and 75% left-handed), and there are two principal pathways in the language network: the dorsal fronto-parietal pathway (which is responsible for articulatory and syntactic tasks) and the ventral temporal pathway (which is responsible for decoding sounds to lexical representations and a word’s significance) [8,9]:

Anterior areas (so called expressive/motor areas) are represented by:

- Broca’s area—the posterior part of the left third frontal gyrus (F3)—Brodmann areas 44 and 45;
- Left insula’s cortex and the underlying white matter;
- Left Rolandic operculum—the lower part of the motor area (Fa);
- Left premotor and prefrontal areas (forward and superior of Broca’s area);
- The supplementary motor area;

Posterior areas (so called comprehensive/sensorial areas) are represented by:

- Wernicke’s area: the posterior part of the first two temporal gyri-T1/T2 (Brodmann area 22);
- The inferior parietal lobes: the angular gyrus (Brodmann area 39), and the supramarginal gyrus (Brodmann area 40);
- The anterior part of the temporal lobe.

### 1.3. Types of Aphasic Syndromes

The most important determining factors of aphasias are the etiology, the site, and the magnitude of the causative lesion/lesions [10]. Age and sex of the patient are also determinant factors for the aphasia secondary to ischemic stroke: younger male patients are more predisposed to develop non-fluent aphasias rather than older patients and female patients.

Taking into consideration the clinical features of aphasia, there are seven types of aphasic syndromes [9,10,11,12]:

- Broca’s aphasia (10–15%);
- Wernicke’s aphasia (15%);
- Conduction aphasia (15%);
- Transcortical aphasias:
  - Transcortical motor aphasia (15–20%);
  - Transcortical sensory aphasia;
  - Mixed transcortical aphasia.
- Global aphasias (24–38%);
- Anomic plus aphasias (20%);
- Atypical aphasias: mixed aphasias, thalamic aphasias, and capsulo-striatal aphasias (10%).

More than 10% of aphasic syndromes remain unclassifiable, particularly in patients with ischemic stroke history.

### 1.4. Etiology of Aphasia

Regarding the etiology of aphasic syndromes, there are various cerebral lesions (acute/chronic, progressive/intermittent/long-lasting, localized/diffuse) that can disturb language networks usually situated in right-handed individuals in the dominant hemisphere (rarely, in the non-dominant hemisphere in right-handed subjects—the so called “crossed aphasia”) [8]. The most commonly involved pathologies that are producing aphasic syndromes are: the cerebro-vascular pathology—“vascular aphasias” (ischemic stroke, hemorrhagic stroke, cerebral veins and dural sinuses thrombosis), post-traumatic brain injuries, brain tumors (left frontal and temporal lobes), cerebral infections (especially viral encephalitis secondary to Herpes simplex virus), neurodegenerative diseases (Alzheimer disease, primary progressive aphasia), multiple sclerosis (rarely). There are also situations when different pathologies reproduce an acute stroke— “stroke mimics”: migraine with aura, focal epilepsy, hypoglycemic coma, encephalopathies (hepatic, uremic, hypoxic, hyponatremic).

## 2. Materials and Methods

**Figure 1:**
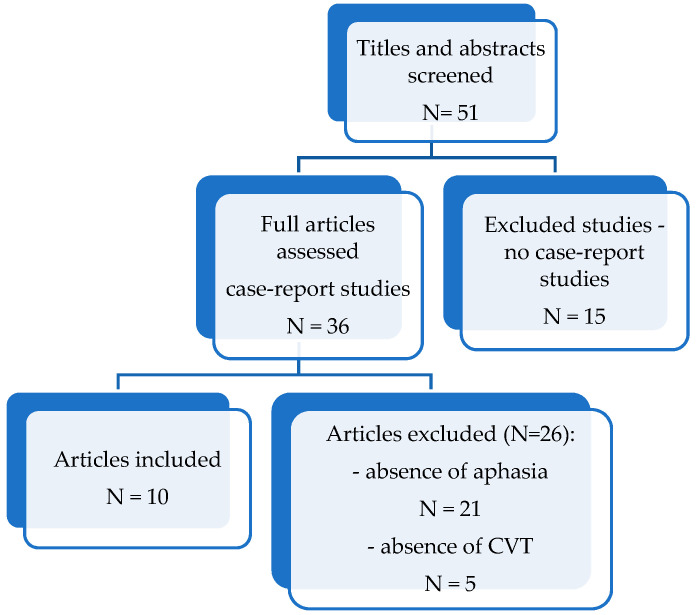
Flowchart of the articles search, N-number of studies.

We achieved a literature search in the PubMed database [cerebral phlebothrombosis aphasia-Search Results-PubMed (nih.gov)], from 2012 to 2022 (in the present), using the following search terms: “aphasia” and “cerebral phlebothrombosis”. Our main objective was to identify the incidence of aphasic syndrome, the main characteristics of those syndromes and correlation between clinical picture and localization of cerebral veins or dural sinuses thrombosis. After identifying the articles, we reviewed the abstracts of the papers and made a preliminary evaluation of their eligibility. After this stage, all potentially eligible papers were accessed and read. We included only those papers which met the eligibility criteria and were relevant to our review (Figure 1).

We applied the following eligibility/including criteria:

(1) Presence of language disturbances (aphasia, dysphasia) and description of aphasia ‘s type or characteristics;
(2) Case-report studies published between 2012–2022;
(3) Adult human studies;
(4) Articles written in English;
(5) The patients included in the study were diagnosed based on an imaging examination: computed tomography (CT), computed tomography venography (CTV), magnetic resonance imaging (MRI), magnetic resonance venography (MRV), intra-arterial angiography (Digital Substraction Angiography—DSA);
(6) Detailed, reliable medical history, physical examination, results of laboratory and imaging examinations were required.

The principal exclusion criteria were the absence of aphasia or lack of information regarding aphasia (N = 21), not being a case-report study (N = 15), absence of CVT and presence of other causes of stroke (N = 5).

## 3. Results

After carefully analyzing all these studies (Table 1), we noted these results.

The first is that the interest for characterizing the distinctive features of aphasic syndromes is extremely poor. The authors usually specify only the term of “dysphasia”, “aphasia” or “language disturbances”, without fitting into a certain aphasia type. This fact explains the difficult identification of eligible articles for our study and consecutively their small number.

The second result is that the most frequent site of CVT that determines aphasia is represented by the left transverse sinus thrombosis associated with a posterior left temporal lesion (due to left temporal cortical veins thrombosis), with a percentage of 90% (*n* = 9/10), followed by the superior sagittal sinus thrombosis associated with a left frontal lesion (due to left frontal cortical veins thrombosis), with a percentage of 40% (*n* = 4/10). Only 20% of cases were presenting isolated cortical veins thrombosis (*n* = 2/10) and 10% (*n* = 1/10) left thalamus lesions due to deep cerebral vein thrombosis. Our results are comparable to recent data from the literature. According to International Study on Cerebral Vein and Dural Sinus Thrombosis, aphasia is commonly the consequence of left transverse sinus thrombosis, followed by the superior sagittal sinus thrombosis [3,13,14].

The third result is that the most frequent type of aphasias found in patients with CVT are: the non-fluent type (Broca’s aphasia and anomic aphasia) with a percentage of 50% (*n* = 5/10) and the global/mixed aphasia (percentage of 30%). Only one case of fluent aphasia (10%, *n* = 1/10) was described in the case-report studies included in our research.

The fourth result is that young female patients (especially in the first 8 weeks postpartum, with/without thrombophilia), oncological cases and severe medical conditions (immune pathologies, toxic substances abuse) are having a higher risk in developing CVT. From our statistical data, the mean age of female patients was 44.42 years (min = 30 years, max = 75 years), meanwhile the mean age of male patients was 48.87 years (min = 27 years, max = 86 years). Regarding the sex incidence, female patients are leaders in CVT (70%, *n* = 7/10), due to their post-partum and post abortion condition.

## 4. Discussion

CVT is a special type of cerebro-vascular pathology. The clinical signs are frequently the consequence of increased intracranial pressure and/or focal brain injury determined by venous infarction or hemorrhage. Venous territories are served by an extensive and widespread network. This is the reason venous territories are less demarcated compared to the arterial territories. CVT can also alter the absorption of the cerebro-spinal fluid (CSF) thru the arachnoid villi. As a result, intracranial pressure increases (with or without cerebral damage), usually in association with superior sagittal sinus (SSS) thrombosis. All these pathophysiological altered mechanisms lead to typical focal neurologic signs and symptoms, depending on the territory of the brain in which the venous drainage is altered, the rapidity of the thrombosis process (immediately or gradually), and the magnitude of brain damage. Superior sagittal sinus (62–80%) and lateral sinus (38–86%) are the most frequently involved sites for thrombosis [3].

Regarding the focal neurologic deficits, specific clinical symptomatology and neurological signs can suggest which area of the brain is damaged. However, the topographic clinical diagnosis of CVT is not as well-defined as in arterial occlusion, frequently being misleading. This characteristic is the consequence of concomitant multiple cerebral veins and dural sinuses thrombosis (more than two-thirds of cases), the numerous anatomic variants of some dural sinuses and some cerebral veins, and the existence of venous collateral circulation [16,25].

When aphasic syndromes are the consequence of dural sinus/sinuses thrombosis or cerebral veins thrombosis, establishing the exact site of lesions might be a challenge. However, the clinical aspects of aphasic syndromes may vary depending on the concomitant occlusion of other dural sinuses or cerebral veins.

1. **Superior Sagittal Sinus (SSS) Thrombosis**

In SSS thrombosis, aphasia is the result of left fronto-parietal hemispheric lesions, secondary to progression of thrombosis to the tributaries bilateral cortical veins [3,16,25,26]. The specific types of aphasias are represented by nonfluent Broca’s aphasia (secondary to left frontal cortical veins thrombosis) and mixed aphasias. Analyzing our results, isolated SSS thrombosis was not found in any of these case-studies. This result strengthens the hypothesis that aphasia is not the result of isolated SSS thrombosis but the consequence of extension of thrombosis to tributaries fronto-parietal cortical veins.

2. **Lateral Sinus (LS) Thrombosis**

Lateral sinus contains two parts: the transverse sinus and the sigmoid sinus, respectively. Regarding the left transverse sinus thrombosis, it is well known that the association of adjacent temporal veins occlusion is the one that produces fluent Wernicke aphasia (40%) [16,25].

Another specific characteristic of this dural sinuses is that the left LS is often hypoplasic; due to this characteristic, after right LS thrombosis, a pseudotumor syndrome is installed, causing bilateral venous drainage deficiency, affecting contralateral structures, especially the inferior part of temporal lobe and cerebellum [25].

In many cases, thrombosis of lateral sinus spreads to other sinuses (especially to SSS) and veins [3,13,26,27]. Analyzing our data, only two case-report studies described aphasic syndromes (one case of non-fluent aphasia and the other one of fluent aphasia) in patients with isolated left LS–transverse part–thrombosis. The other cases of LS thrombosis were accompanied by other sinuses or cerebral veins thrombosis (*n* = 7/10).

3. **Isolated Cortical Veins Thrombosis**

Without association with dural sinus thrombosis, isolated thrombosis of cortical veins is considered a rare affection (2%) [16,25]. A plausible explanation would be that it is frequently underdiagnosed, due to imaging barriers, being identified with difficulty using the traditional MRI sequences and MRV [16,25]. The most common localization of occlusion is at the levels of the superior cortical veins, producing nonfluent Broca’s aphasia or mixed aphasias [25]. From our collected data, there is only one case of isolated left cortical veins thrombosis.

4. **Deep Cerebral Veins Thrombosis**

Deep venous system thrombosis can be suspected in patients with altered state of consciousness (diffuse encephalopathy or even coma) and motor deficits (bilateral or fluctuating alternating paresis) [3,26]. Deep cerebral veins thrombosis (vein of Galen, basal veins of Rosenthal, internal cerebral veins, straight sinus) produces damages into the caudate nucleus and thalami, being present in almost 18% of patients diagnosed with CVT [16]. In rare situations, benign cases of thrombosis of the deep cerebral veins were noted producing left thalamic lesions with mixed transcortical aphasia [16].

Over the last few years, several case reports have brought to attention the small incidence of aphasia in the clinical picture of CVT. Unfortunately, few of those studies made a precise evaluation of the aphasic syndrome and a clinical-imagistic correlation.

Alexander Engelmann and his collaborators [20] published in 2021 an interesting case-report of an 86-year-old right-handed male with a complex oncological and neurosurgical pathology (history of colon adenocarcinoma, surgically treated, and recent surgery for right sphenoid wing meningioma), which presented in their emergency department with several transient episodes of fluent aphasia, during at most 10 min, being accompanied by one episode of involuntary right-handed grip, being initially considered as a TIA, taking into consideration that the symptomatology completely remitted. Although the Non-contrast head CT, CT Angiography head and neck, and perfusion CT did not highlight any sign of arterial occlusion or perfusion defects, they revealed the absence of opacification of the left transverse and sigmoid sinuses. They continued the investigations, and after performing the brain MRI with gadolinium, they found a new left transverse sinus thrombus. After 13 days of anticoagulation (LMWH) the patient did not experience any of the previous symptoms.

Giovana Ennis and her team [18] brought to attention another interesting and also a complex case of a 76-year-old woman (history of immune thrombocytopenic purpura, treated at that time with prednisolone, arterial hypertension, atrial fibrillation, hypothyroidism and pulmonary embolism), which presented in the emergency department complaining of amnesia and lethargy that had started a week earlier and were becoming more frequent, multiple episodes of transient right hemiparesis in the last two days, with a duration of 1–2 h. At the admission, the neurological examination revealed a 2/5 MRC right sided hemiparesis and global aphasia. The patient underwent cerebral computed tomography (CT) that excluded ischemic or hemorrhagic vascular event or any space-occupying process. After these examinations, the patient was re-examined, and, surprisingly, aphasia had disappeared, but a slight (4/5) right hemiparesis remained. Five hours later, her clinical status deteriorated, again presenting global aphasia and 3/5 MRC right hemiparesis. They decided to perform a CT angiography, which did not bring any pathological signs, excluding intracranial arterial occlusion or stenosis. On the first day after the admission in the Stroke Unit, the patient suffered three tonic-clonic seizures, with involuntary movements in the right limbs. Twelve hours later, a new cerebral CT was performed and revealed a vascular lesion situated in the left Rolandic cortico-subcortical region, with a hemorrhagic component and reduced amplitude of the regional sulci. Taking into consideration the high density of the medial third of the superior longitudinal sinus (SSS), cerebral veno-CT was immediately performed, which confirmed the lack of opacification of the superior longitudinal sinus and the right lateral sinus. The presence of the left fronto-parietal intraparenchymatous lesion was evocative for a venous infarct. She was also treated with LMWH, adjusted to the hematological pathology.

Another recent study conducted by Ayele et al. [15], reported the case of a 30-year-old right-handed Ethiopian female patient, who gave birth to a stillborn fetus 2 months previously, which presented with resistant to usual medications headache, accompanied by word-finding difficulty, nausea, and blurred vision, with a 2-week onset. At the neurological examination there were no focal deficits or cranial nerves signs. The language assessment showed normal fluency, compression, repetition, and reading. At the naming probe (assessed using a word generating test of 60 s), she has been found with difficulties, indicating anomia. The examination of fundus showed bilaterally papilledema. Brain MRI showed left temporo-parietal ischemia. MR venography showed thrombosis of the left transverse, sigmoid sinus, and corresponding cortical veins. During their research, they found only two similar cases, described by Kuan et al. (2014) [23], which symptoms were the consequence of left transverse sinus, sigmoid sinus and internal jugular vein thrombosis, with parenchymal hemorrhages in the left temporo-parietal region with effacement of the left cerebral sulci, and Sarma et al. (2004) [15], which symptoms were the consequence of superior, inferior, straight sinuses and also vein of Galen thrombosis.

In 2014, Tuncel and his collaborators [24] reported the case of a young patient (a 27-year-old male), diagnosed with advanced-stage small cell lung cancer two months previously, which presented to the Department of Medical Oncology, with Broca’s aphasia and general seizures. The MRI of the brain did not show any pathological features. After initiating the chemotherapy, on day six of the second cycle, the patient presented in the emergency department 4 h succeeding the onset of a stroke. Using the MRI scan, they revealed a left transverse sinus, as well as a left sigmoid sinus thrombosis, accompanied by acute left parietal venous infarction.

In 2021, Jianu et al. [16], handled a case, that was both difficult and interesting, of a 38-year-old woman with pre-existing hypertension, on her 18th postpartum day, which was presented in the emergency department accusing sudden onset of severe headache for 2 days, accompanied by moderate right hemiparesis (4/5 MRC) and language disturbances (she was unable to speak or to understand orders properly, although the repetition of words and sentences was possible–mixed transcortical aphasia). All of these symptoms can be explained by the localization of the left cerebral deep venous infarcts (affecting the caudate, putamen, left thalamus, and periventricular white matter-internal capsule) that she presented [16].

Searching into the specialized literature, we observed a small number of observational studies which followed the clinical features of aphasic patience with CVT. The VENOST study [28] is the latest and the largest retrospective, prospective, multicenter, hospital-based, observational study that followed the records of 1144 patients with CVT, collected between 2000 and 2013 from patients’ medical files. Between 2013 and 2015 the patients were included prospectively. Statistical data showed that the percentage of aphasia or dysarthria among all those patients who suffered from CVT was only 1.2% [28].

Analyzing all this data, we can easily observe that it is hard to make a statistically significant hypothesis regarding the incidence of different types of aphasic syndromes in CVT, taking into consideration that the diagnosis of a certain type of aphasia requires specific assessments, using approved tests, while the patient is cooperating (patients frequently have reduced arousal). However, some conclusions are clear from this study:

- aphasia is commonly the consequence of concomitant left transverse sinus thrombosis with left cortical temporal veins, followed by the superior sagittal sinus thrombosis with extension into tributary fronto-parietal cortical veins;
- depending on the site and the size of the brain damage (cerebral vasogenic/cytotoxic edema, venous infarction, intracranial hypertension), the most frequent types of aphasias are: non-fluent aphasias-Broca’s aphasia and anomic plus aphasia (if the lesions are situated in the anterior language areas), fluent aphasias-Wernicke’s aphasia, transcortical sensory aphasia (if the lesions are situated in the posterior language areas), mixed or global aphasias (in the cases of larger lesions);
- in LS sinus thrombosis (the transversal portion) associated with left cortical temporal veins thrombosis, the most common type of aphasia is Wernicke’s aphasia (40%) [16];
- in many cases, the LS thrombosis spreads to the SSS, symptoms and signs of SSS thrombosis depending on the involvement of cerebral veins and other dural sinuses. The most often involved are the superior cerebral veins (Rolandic, parieto-occipital and posterior temporal) which empty into the SSS. If the thrombosis spreads to the deep veins system, altering the Ascending Reticular Activating System (ARAS), awakening alteration may occur;
- the clinical picture depends on the location and the dimensions of cerebral lesion: extensive lesions can determine global aphasia, or mixed transcortical aphasias; meanwhile smaller lesions might determine Wernicke’s aphasia, transcortical sensory aphasia, Broca aphasia or anomic aphasia.

Our small study managed to highlight the small amount of information that has been gathered until now regarding the characteristics of aphasic syndromes in CVT, and this might be considered a great limitation. Unfortunately, the small number of cases described in the literature made difficult our intention to demonstrate a certain pattern of language disturbances, using the localization of the CVT. Mostly, the greatest impediment was the fact that few authors focused their attention on the clinical characteristics of aphasic syndromes, analyzing and describing the clinical aspects of language disturbances in an incomplete manner. However, this might be the beginning of a new research theme in this rare and underdiagnosed condition. We aim to continue our study and to gather further information over the next years to support us in outlining a clinical template that will guide clinicians in establishing the diagnosis of CVT much faster, more easily, and more accurately.

## 5. Conclusions

In conclusion, the early identification of aphasia in CVT can help us to provide a better prognosis of patients and the site of the lesion and guide us in the differential diagnosis of this cerebro-vascular pathology. Although aphasia is a rare syndrome in a rare disease (CVT), we should look toward new clinical data regarding this issue, insisting over the examination of the patients’ language and trying to determine whether, the presence of aphasia might be a severity marker as it is considered in stroke. We should carefully look after patients with associated conditions that have fully demonstrated their potentially prothrombotic characteristic, especially in young women (postpartum/post abortion) and patients with inherited thrombophilia.

What is worth underlying is that aphasic syndromes are not the consequence of thrombosis of a specific, isolated dural sinus, but the result of thrombosis extension to cortical veins or other dural sinuses (especially the left LS and the SSS). Venous infarction in strategic language areas is also the main determining cause of aphasic syndromes.

## Figures and Tables

**Table 1:** Distribution on age, gender, associated pathologies, site of thrombosis and types of aphasia in CVT patients-case-report studies 2012–2022 Pub-Med data [15,16,17,18,19,20,21,22,23,24].

Study	First Author and Year of Publication	Age	Gender	Associated Pathologies	Site of CVT	Type of Aphasia
*Reversible anomia and cerebral venous thrombosis: A case report and review of the literature*	Biniyam A. Ayele et al. (2022) [15]	30	F	two months after dead fetus birth	left transverse, sigmoid sinus (lateral sinus), corresponding cortical veins.	non-fluent aphasia, anomic aphasia
*Diagnosis and Management of Mixed Transcortical Aphasia Due to Multiple Predisposing Factors, including Postpartum and Severe Inherited Thrombophilia, Affecting Multiple Cerebral Venous and Dural Sinus Thrombosis: Case Report and Literature Review*	Dragos, Catalin Jianu et al. (2021) [16]	38	F	inherited thrombophilia 18 days postpartum	superior sagittal sinus, the straight sinus, the vein of Galen, the deep venous system on the left, the lateral sinus left internal jugular vein	mixed transcortical aphasia (isolation aphasia)
*Bilateral thalamic lesion presenting as Broca’s type subcortical aphasia in cerebral venous thrombosis: Index case report*	Shambaditya Das et al. (2021) [17]	35	M	multiple substances abuse (alcohol, tobacco, and cannabis), thrombophilia—decreased levels of protein C, protein S and antithrombin III	superior sagittal sinus bilateral transverse sinus (lateral sinus)	non-fluent aphasia–Broca‘s aphasia
*Cerebral Venous Thrombosis and Its Clinical Diversity*	Giovana Ennis et al. (2021) [18]	75	F	immune thrombocytopenic purpura, arterial hypertension, and pulmonary embolism	superior sagittal sinus the left lateral sinus	global aphasia
*Cerebral venous sinus thrombosis associated with spontaneous heparin-induced thrombocytopenia syndrome after total knee arthroplasty*	Steven R Hwang et al. (2020) [19]	56	F	hypertension degenerative osteoarthritis	left transverse and sigmoid sinuses (lateral sinus) left internal jugular vein	unspecified type
*The ugly duckling of aphasia: cerebral venous sinus thrombosis as a mimic of TIA and stroke*	Alexander Engelmann et al. (2020) [20]	86	M	colon adenocarcinoma (status post resection); recent surgery for right sphenoid wing meningioma	left transverse (lateral) sinus	fluent aphasia
*Bilateral corpus callosum and corona radiata infarction due to cerebral venous sinus thrombosis presenting as headache and acute reversible aphasia: A rare case report*	Rui Lan et al. (2020) [21]	30	F	20 days post-partum	superior sagittal sinus the left transverse (lateral) sinus	non-fluent aphasia–Broca‘s aphasia
*Ipsilateral Dural Thickening and Enhancement: A Sign of Isolated Cortical Vein Thrombosis? A Case Report and Review of the Literature*	Davide Marco Croci et al. (2016) [22]	30	F	14 days postpartum	left cortical veins thrombosis	global aphasia
*Anomia and mild headache: A subtle presentation of cerebral venous thrombosis*	WS Kuan (2014) [23]	52	F	thrombophilia: low levels of protein C activity of 64% expected range 70–130%), protein S activity of 50% (expected range 55–140%) and anti-thrombin III level of 62% (expected range 80–120)	left transverse and sigmoid (lateral) sinuses, the left internal jugular vein	non-fluent aphasia, anomic aphasia
*Broca‘s aphasia due to cerebral venous sinus thrombosis following chemotherapy for small cell lung cancer: A case report and review of literature*	Tolga Tuncel et al. (2014) [24]	27	M	advanced-stage small cell lung cancer cisplatin-based chemotherapy	left transverse (lateral) sinus thrombosis	non-fluent aphasia, anomic aphasia

## Data Availability

Not applicable.

## References

[1] Ferro J.M., Canhaão P., Stam J., Bousser M.-G., Barinagarrementeria F. (2004). Prognosis of cerebral vein and dural sinus thrombosis: Results of the International Study on Cerebral Vein and Dural Sinus Thrombosis (ISCVT). Stroke.

[2] Sparaco M., Feleppa M., Bigal M.E. (2015). Cerebral Venous Thrombosis and Headache-A Case-Series. Headache.

[3] Jianu D.C., Jianu S.N., Munteanu G., Dan F.T., Bârsan C. (2018). Cerebral Vein and Dural Sinus Thrombosis. Ischemic Stroke of Brain.

[4] Bousser M.-G., Ferro J.M. (2007). Cerebral venous thrombosis: An update. Lancet Neurol..

[5] Devasagayam S., Wyatt B., Leyden J., Kleinig T. (2016). Cerebral Venous Sinus Thrombosis Incidence Is Higher Than Previously Thought: A retrospective population-based study. Stroke.

[6] Damak M., Crassard I., Wolff V., Bousser M.-G. (2009). Isolated Lateral Sinus Thrombosis: A series of 62 patients. Stroke.

[7] Ferro J., Lopes M.G., Rosas M., Ferro M., Fontes J. (2002). Long-Term Prognosis of Cerebral Vein and Dural Sinus Thrombosis: Results of the VENOPORT study. Cerebrovasc. Dis..

[8] Jianu D.C., Jianu S.N., Petrica L., Dan T.F., Munteanu G., Sanchetee P. (2021). Vascular Aphasias. Ischemic Stroke.

[9] Abou Zeki D., Hillis A., Masud H., Schott J.M. (2016). Acquired Disorders of Language and Speech. Oxford Textbook of Cognitive Neurology and Dementia.

[10] Croquelois A., Godefroy O., Godefroy O. (2013). Vascular Aphasias. The Behavioral and Cognitive Neurology of Stroke.

[11] Goodglass H., Kaplan E. (1983). The Assessment of Aphasia and Related Disorder.

[12] Swanberg M.M., Nasreddine Z.S., Mendez M.F., Cummings J.L., Christopher G. (2007). Speech and Language. Goetz, Textbook of Clinical Neurology.

[13] Ferro J.M., Canhão P., Grotta J.C., Albers G.W., Broderick J.P., Kasner S.E., Lo E.H., Mendelow A.D., Sacco R.L., Wong L.K.S. (2016). Cerebral Venous Thrombosis. Stroke (Pathophysiology, Diagnosis, and Management).

[14] Einhäupl K., Bousser M.G., de Bruijn S.F., Ferro M., Martinelli I., Masuhr F., Stam J. (2006). FEFNS guideline on the treatment of cerebral venous sinus thrombosis. Eur. J. Neurol..

[15] Ayele B.A., Abdella R.I., Wachamo L.Z. (2022). Reversible anomia and cerebral venous thrombosis: A case report and review of the literature. J. Med. Case Rep..

[16] Jianu D., Jianu S., Dan T., Iacob N., Munteanu G., Motoc A., Băloi A., Hodorogea D., Axelerad A., Pleș H. (2021). Diagnosis and Management of Mixed Transcortical Aphasia Due to Multiple Predisposing Factors, including Postpartum and Severe Inherited Thrombophilia, Affecting Multiple Cerebral Venous and Dural Sinus Thrombosis: Case Report and Literature Review. Diagnostics.

[17] Das S., Dubey S., Pandit A., Ray B.K. (2021). Bilateral thalamic lesion presenting as Broca’s type subcortical aphasia in cerebral venous thrombosis: Index case report. BMJ Case Rep..

[18] Ennis G., Domingues N., Marques J.S., Ribeiro P., Andrade C. (2021). Cerebral Venous Thrombosis and Its Clinical Diversity. Cureus.

[19] Hwang S.R., Wang Y., Weil E.L., Padmanabhan A., Warkentin T.E., Pruthi R.K. (2021). Cerebral venous sinus thrombosis associated with spontaneous heparin-induced thrombocytopenia syndrome after total knee arthroplasty. Platelets.

[20] Engelmann A., DiPastina K., Liu T. (2021). The ugly duckling of aphasia: Cerebral venous sinus thrombosis as a mimic of TIA and stroke. J. Community Hosp. Intern. Med. Perspect..

[21] Lan R., Ma Y.Z., Shen X.M., Wu J.T., Gu C.Q., Zhang Y. (2020). Bilateral corpus callosum and corona radiata infarction due to cerebral venous sinus thrombosis presenting as headache and acute reversible aphasia: A rare case report. BMC Neurol..

[22] Croci D.M., Michael D., Kahles T., Fathi A.R., Fandino J., Marbacher S. (2016). Ipsilateral Dural Thickening and Enhancement: A Sign of Isolated Cortical Vein Thrombosis? A Case Report and Review of the Literature. World Neurosurg..

[23] Kuan W.S. (2014). Anomia and Mild Headache: A Subtle Presentation of Cerebral Venous Thrombosis. Hong Kong J. Emerg. Med..

[24] Tuncel T., Ozgun A., Emirzeoğlu L., Celiïk S., Demiïr S., Bilgi O., Karagoz B. (2014). Broca’s aphasia due to cerebral venous sinus thrombosis following chemotherapy for small cell lung cancer: A case report and review of literature. Oncol. Lett..

[25] Jianu D.C., Jianu S.N., Dan T.F., Munteanu G., Copil A., Birdac C.D., Motoc A.G.M., Axelerad A.D., Petrica L., Arnautu S.F. (2022). An Integrated Approach on the Diagnosis of Cerebral Veins and Dural Sinuses Thrombosis (a Narrative Review). Life.

[26] Ulivi L., Squitieri M., Cohen H., Cowley P., Werring D.J. (2020). Cerebral venous thrombosis: A practical guide. Pract. Neurol..

[27] Bousser M.G., Barnett H.J.M., Mohr J.P., Choi D.W., Grotta J.C., Weir B., Wolf P.A. (2004). Cerebral Venous Thrombosis. Stroke (Pathophysiology, Diagnosis, and Management).

[28] Duman T., Uluduz D., Midi I., Bektas H., Kablan Y., Goksel B.K., Milanlioglu A., Orken D.N., Aluclu U., Colakoglu S. (2017). A Multicenter Study of 1144 Patients with Cerebral Venous Thrombosis: The VENOST Study. J. Stroke Cerebrovasc. Dis..

